# TRAILBLAZER‐ALZ 4: A phase 3 trial comparing donanemab with aducanumab on amyloid plaque clearance in early, symptomatic Alzheimer's disease

**DOI:** 10.1002/alz.70293

**Published:** 2025-05-19

**Authors:** Stephen Salloway, Andrew Pain, Elly Lee, Michelle Papka, Margaret B. Ferguson, Hong Wang, Haoyan Hu, Ming Lu, Ena Oru, Paul A. Ardayfio, Deirdre B. Hoban, Emily C. Collins, Dawn A. Brooks, John R. Sims

**Affiliations:** ^1^ Department of Neurology and Department of Psychiatry Alpert Medical School of Brown University Providence Rhode Island USA; ^2^ Butler Hospital Providence Rhode Island USA; ^3^ Eli Lilly and Company Indianapolis Indiana USA; ^4^ Irvine Clinical Research Irvine California USA; ^5^ The Cognitive and Research Center of New Jersey LLC Springfield New Jersey USA

**Keywords:** aducanumab, amyloid clearance, amyloid‐related imaging abnormalities, amyloid‐targeting therapies, donanemab, infusion‐related reaction

## Abstract

**INTRODUCTION:**

The phase 3, open‐label TRAILBLAZER‐ALZ 4 study compared the effect of donanemab versus aducanumab on amyloid plaque (AP) clearance in participants with early symptomatic Alzheimer's disease.

**METHODS:**

Participants (*n* = 148) were randomized 1:1 to receive intravenous donanemab (700 mg every 4 weeks for three doses, then 1400 mg every 4 weeks thereafter) or aducanumab (per label). AP was measured with florbetapir F 18 positron emission tomography. AP clearance was defined as < 24.1 Centiloids.

**RESULTS:**

At 6, 12, and 18 months, AP clearance was achieved in 37.9%, 70.0%, and 76.8%, respectively, of donanemab‐treated participants versus 1.6%, 24.6%, and 43.1% of aducanumab‐treated participants (*P *< 0.001). Median time to clearance was 359 versus 568 days for donanemab‐ versus aducanumab‐treated participants (*P *< 0.001). Amyloid‐related imaging abnormality (ARIA)‐edema/effusion occurred in 23.9% and 34.8% of donanemab‐ and aducanumab‐treated participants, respectively.

**DISCUSSION:**

Donanemab treatment resulted in earlier and greater AP clearance compared to aducanumab. ARIA frequencies were consistent with prior studies.

Clinical trial registration: No: NCT05108922, TRAILBLAZER‐ALZ 4

**Highlights:**

- Here we report the first direct comparator study of two amyloid‐targeting therapies.
- This was the first investigation of donanemab on biomarker efficacy regardless of tau levels.
- Donanemab demonstrated superiority over aducanumab in amyloid plaque (AP) clearance.
- The depth and speed of AP removal did not affect amyloid‐related imaging abnormality risk or incidence.

## 1 BACKGROUND

In the amyloid cascade, the accumulation of amyloid beta (Aβ) peptide in the form of amyloid plaques (APs) in the brain is an early and necessary event in Alzheimer's disease (AD), leading to downstream events including tau pathology, synaptic loss, and neuronal death resulting in clinical symptoms of cognitive impairment and eventually dementia.^1^, ^2^ Given that an abnormal level of Aβ is one of the key pathological hallmarks of AD, as defined by the 2018 National Institute on Aging–Alzheimer's Association (NIA‐AA) research framework,^3^ Aβ has been a major target and focus of AD research.

Several phase 3 trials that are ongoing (recruiting or active) or have recently been completed aim to assess the efficacy of amyloid‐targeting therapies (ATTs). The therapeutic agents aducanumab, donanemab, and lecanemab each target different epitopes or have different binding affinities to the various forms of Aβ.^4^, ^5^, ^6^, ^7^ Although there has been mixed success of these therapies to remove amyloid, a new era of ATTs has begun with the regulatory approvals of aducanumab in 2021, lecanemab in 2023, and donanemab in 2024.

Aducanumab is a human amyloid‐targeting monoclonal antibody that selectively targets aggregated forms of Aβ, including soluble oligomers and insoluble fibrils.^8^ The efficacy of aducanumab has previously been described. The EMERGE phase 3 trial of aducanumab demonstrated a significant reduction in clinical decline, while the ENGAGE phase 3 study did not replicate this finding.^9^ The US Food and Drug Administration granted accelerated approval for clinical use of aducanumab based on its capability to reduce AP levels and because of the need for disease‐modifying therapies at that time.^10^ However, in January 2024, the manufacturer of aducanumab chose to discontinue the development and commercialization of aducanumab globally.^11^ Donanemab is a humanized immunoglobulin G1 monoclonal antibody that selectively binds the insoluble N‐terminal truncated form of Aβ (third amino acid, pyroglutamate formation [N3pG]) that is only present in mature Aβ plaques in the brain. The Aβ‐bound donanemab complex is cleared by microglial‐mediated phagocytosis.^12^ In the phase 3 TRAILBLAZER‐ALZ 2 study, treatment with donanemab reduced brain AP levels that resulted in slowing of clinical progression at the end of 18 months^13^ as a clinically meaningful benefit, replicating the results of the phase 2 TRAILBLAZER‐ALZ study.^14^

Although both donanemab and aducanumab have demonstrated amyloid reduction and potential clinical benefits,^9^, ^13^, ^14^ indirect comparisons on amyloid‐lowering outcomes between ATTs are not advised due to differences in the study population and trial designs. Herein, we report the results of TRAILBLAZER‐ALZ 4 (NCT05108922), a phase 3, open‐label, active‐comparator study that assessed the superiority of donanemab versus aducanumab on brain AP clearance in participants with early symptomatic AD.

## 2 METHODS

### 2.1 Trial conduct and oversight

The TRAILBLAZER‐ALZ 4 study was a 76‐week, randomized, open‐label, parallel, multicenter, active‐comparator, phase 3 trial conducted at 31 sites in the United States. The study was initiated on September 24, 2021, and was completed on September 19, 2023. The trial was conducted in accordance with the Declaration of Helsinki, the International Council for Harmonisation of Technical Requirements for Pharmaceuticals for Human Use Good Clinical Practice guidelines, and local regulatory requirements. The study protocol and statistical analysis plan were approved by an independent ethics committee or institutional review board at each study site. All participants provided written informed consent before the start of the study.

### 2.2 Trial design and participants

The trial included participants 50 to 85 years of age who had early symptomatic AD (mild cognitive impairment^15^ or AD with mild dementia^3^). Eligible participants had a Mini‐Mental State Examination (MMSE) score of 20 to 30, Clinical Dementia Rating Global Score of 0.5 or 1.0, and evidence of amyloid pathology as assessed by florbetapir 18 F positron emission tomography (PET) imaging (positive visual scan and ≥ 37 Centiloids [CL] or negative visual scan and ≥ 50 CL). Baseline flortaucipir 18 F PET imaging was performed but not used for eligibility purposes.

Key exclusion criteria included the presence of amyloid‐related imaging abnormalities (ARIAs) of edema/effusion (ARIA‐E), more than four cerebral microhemorrhages, more than one area of superficial siderosis, and any intracerebral hemorrhage > 1 cm or severe white matter disease at screening. Participants were also excluded from the study if they had a history of bleeding disorder or use of medications with platelet antiaggregant or anticoagulant properties (aspirin at ≤ 325 mg daily was an exception to be consistent with prior aducanumab clinical trials). A full list of inclusion/exclusion criteria can be found at ClinicalTrials.gov/study/NCT05108922.

Participants who met the eligibility criteria were randomly assigned in a 1:1 ratio to receive either open‐label donanemab once every 4 weeks (700 mg for the first three doses and 1400 mg thereafter) or aducanumab according to the US package insert (ADUHELM [aducanumab‐avwa])^10^ via intravenous infusion. For between‐group comparability, participant randomization was stratified by amyloid level and apolipoprotein E (*APOE*) ε4 status (non‐carrier/heterozygous/homozygous). Donanemab‐treated participants who met donanemab cessation criteria discontinued further infusions but continued all other assessments for the remaining duration of the open‐label period. Achievement of stopping criteria was assessed by florbetapir PET (performed at 24 and 52 weeks) and defined as an AP level < 11 CL on any one scan or ≥ 11 to < 25 CL on two consecutive scans. Change in AP level was assessed with florbetapir PET by personnel blinded to treatment assignment. Florbetapir PET images were processed using established pipelines. The 5 minute frames were motion corrected and averaged to create a static image. Each static image was then spatially aligned to the standard brain template, and the mean cortical standardized uptake value ratio with a whole cerebellar reference region was measured^16^ and converted to CL.

**RESEARCH IN CONTEXT**

1. **Systematic review**: Although multiple amyloid‐targeting therapies (ATTs) have been shown to reduce amyloid plaque (AP) in the brain, comparing results in the absence of active‐comparator trials is challenging due to differences in study populations and clinical trial designs.
2. **Interpretation**: In this first active‐comparator study between two ATTs, donanemab showed superiority over aducanumab in AP clearance, and results revealed that the speed and depth of amyloid removal did not have a relationship with increased risk/incidence of amyloid‐related imaging abnormalities.
3. **Future directions**: Donanemab will continue to be assessed and monitored in clinical practice. Ongoing and future studies will investigate AP removal in preclinical Alzheimer's disease and explore different dosing methods.

### 2.3 Outcomes/endpoints

The two coprimary outcomes assessed in participants with early symptomatic AD were the superiority of donanemab versus aducanumab as assessed by the proportion of participants to reach AP clearance (< 24.1 CL)^17^, ^18^ using florbetapir PET at 6 months in (1) the overall population (including the low–medium tau subpopulation and other tau subpopulations) and (2) the low–medium tau subpopulation. The low–medium tau subpopulation was defined by visual and quantitative reads as previously described.^13^, ^14^, ^19^, ^20^ The key secondary outcomes were the superiority of donanemab versus aducanumab in the time to reach AP clearance and in the mean absolute change from baseline in brain AP level at 6, 12, and 18 months. Safety outcomes included spontaneously reported adverse events (AEs), clinical laboratory test results, body‐weight measurements (only for participants receiving aducanumab, per weight‐based dosing),^10^ findings on locally read 12‐lead electrocardiography (at screening), physical and neurological examinations, centrally read magnetic resonance imaging (MRI), and the Columbia–Suicide Severity Rating Scale score. ARIA‐E, ARIA of microhemorrhage and hemosiderin deposits (ARIA‐H), and infusion‐related reactions were AEs of special interest because they were considered class effects or observed in previous trials.^13^, ^14^, ^21^, ^22^ To comprehensively evaluate ARIAs, treatment‐emergent AE (TEAE) cluster and MRI data were used to identify ARIA cases. The TEAE cluster for ARIA‐E included ARIAs of edema/effusion, brain edema, and vasogenic cerebral edema. The TEAE cluster for ARIA‐H included ARIAs of microhemorrhage and hemosiderin deposits, brainstem microhemorrhage, cerebellar microhemorrhage, cerebral hemosiderin deposit, cerebral microhemorrhage, and superficial siderosis of the central nervous system. A full list of safety assessments can be found at clinicaltrials.gov/study/NCT05108922.

### 2.4 Statistical analyses

Statistical analyses were performed on the following predefined analysis sets: (1) the overall population, defined as all randomized participants with a baseline and at least one postbaseline florbetapir PET result; (2) the low–medium tau subpopulation, defined as all participants in the overall population with a baseline flortaucipir PET who met the low–medium tau criteria; and (3) the safety analysis set, defined as all participants who were exposed to study treatment.

Approximately 200 participants were planned to be enrolled and randomized in a 1:1 ratio. It was further anticipated that approximately 50% of participants randomized would belong to the low–medium tau subpopulation. This sample size was determined to provide at least 98% power to demonstrate that donanemab is superior to aducanumab in achieving amyloid brain plaque clearance individually at 6 months in both the overall population and the low–medium tau subpopulation. Power estimates were obtained using specific contrasts for the 6 month time point of the treatment‐by‐time interaction effect within the framework of the generalized linear mixed‐effect model, assuming a logit link. The assumptions for this power calculation were that 35.7%, 50.1%, and 54.6% of donanemab‐treated participants would reach AP clearance at 6, 12, and 18 months, respectively. The corresponding AP clearance proportions for aducanumab‐treated participants were assumed to be 3.0%, 22.8%, and 27.8%, respectively. The simulation for the power calculation and sample size determination was performed in SAS version 9.4 (SAS Institute Inc.), and all sample size estimates were obtained by assuming a two‐sided significance level of 0.05.

The prespecified comparison of donanemab to aducanumab in AP clearance (defined as < 24.1 CL as measured by florbetapir PET^13^, ^14^, ^17^, ^18^) at 6 months was performed using logistic regression. The logistic regression model included the treatment, *APOE* ε4 carrier status, baseline amyloid level, and baseline age as fixed effects. Treatment effects from these logistic regression models were summarized using estimated probabilities within each treatment group, along with odds ratios and 100*(1−α)% confidence intervals (CIs), where α was the two‐sided significance level for the primary endpoints.

For the prespecified analyses of mean change at 6, 12, and 18 months, change from baseline was defined as the PET CL value at the specific time point minus the baseline PET CL value. Mean percent change from baseline in brain AP was defined as the PET CL value at the specific time point minus the baseline PET CL value, divided by the baseline PET CL value. For the prespecified analysis of time to AP clearance, participants were categorized as either meeting or not meeting the criterion for AP clearance based on florbetapir F18 PET CL values or not meeting this criterion. Time to event was defined as the difference between the date of the PET scan that showed AP clearance and the date of the first dose of study treatment. Participants who did not meet this criterion were censored at the time of their final PET scan. As there was only one postbaseline PET assessment within the first 6 months of the trial, mean change from baseline in AP levels at 6 months was analyzed using an analysis of covariance model with the treatment group as the fixed‐effect variable of interest and *APOE* ε4 carrier status, baseline amyloid level, and baseline age as covariates. For the prespecified analysis of mean change from baseline at 12 and 18 months, a separate mixed model for repeated measures (MMRM) analysis with data up to and including that specific time point was used. This model included the treatment, time, and treatment‐by‐time interaction as fixed effects and the *APOE* ε4 carrier status, baseline amyloid level, and baseline age as covariates. Mean percent change from baseline was modeled with the same approach as that outlined for the mean change analyses. Time to AP clearance between the two treatment groups was analyzed and compared using a log‐rank test for survival data. Kaplan–Meier estimates of the proportion of participants reaching AP clearance and the *P* value from the log‐rank test were used to determine the superiority of donanemab versus aducanumab in lowering time to AP clearance.

For the prespecified assessments relating to the low–medium tau subpopulation, the statistical models were identical to those of the overall population, with analysis of covariance applied to the 6‐month data and an MMRM analysis applied to the 12‐ and 18‐month data. Both primary and key secondary endpoints were adjusted for multiplicity. If both co‐primary endpoints were statistically significant, key secondary endpoints were evaluated in a gated manner as follows: mean change from baseline in AP levels at 6 months, then at 12 months, the time to AP clearance was tested at study completion, and the mean change from baseline in AP level at 18 months.

Exploratory biomarker samples, including neurofilament light chain (NfL) and plasma tau analyses, were collected throughout the study. Prespecified analyses were performed in both the overall study population and the low–medium tau subpopulation. Biomarker values were log‐transformed for analyses. An MMRM analysis was used to compare the change from baseline to scheduled collection times in the overall population. The model included the treatment, visit, and treatment‐by‐visit interaction as fixed categorical effects and the baseline biomarker level and baseline age as continuous effects.

AEs were evaluated in the safety analysis set and according to event frequency.

Statistical analyses were performed using SAS version 9.4. Further details regarding statistical analyses can be found at ClinicalTrials.gov/study/NCT05108922.

## 3 RESULTS

### 3.1 Trial population and baseline characteristics

**FIGURE 1:**
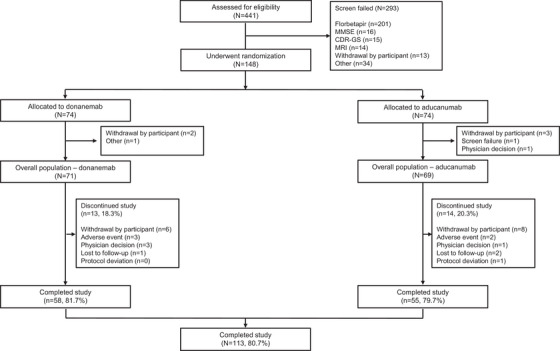
CONSORT diagram. CDR‐GS; Clinical Dementia Rating Scale Global Score; CONSORT, Consolidated Standards of Reporting Trials; MMSE, Mini‐Mental State Examination; MRI, magnetic resonance imaging

Of the 441 patients assessed for eligibility, 148 were enrolled in the trial and randomly assigned 1:1 to receive donanemab or aducanumab (Figure 1). In the donanemab group, the mean (standard deviation [SD]) age was 74.1 (6.9) years, 53.5% were female, and 69.0% were *APOE* ε4 carriers. In the aducanumab group, the mean (SD) age was 72.7 (6.8) years, 60.9% were female, and 71.0% were *APOE* ε4 carriers. The mean (SD) baseline MMSE score was 25.0 (2.7) in the donanemab group and 24.4 (2.9) in the aducanumab group. The mean (SD) AP level on florbetapir PET was 97.2 (29.2) CL and 102.3 (34.6) CL in the donanemab and aducanumab groups, respectively. The mean (SD) AD‐signature weighted neocortical standardized uptake value ratio using flortaucipir PET was 1.3 (0.3) in the donanemab group and 1.3 (0.3) in the aducanumab group. Relative to the overall population, the low–medium tau subpopulation was numerically comparable in age, sex, and *APOE* ε4 carrier status. Participants in the low–medium tau subpopulation had a similar baseline MMSE score and AP level to those in the overall population, but they had a lower baseline tau level (Table 1).

**TABLE 1:** Baseline demographics and clinical characteristics.

	Overall population (*n* = 140)		Low–medium tau subpopulation (*n* = 55)	
Demographic/scale	Donanemab (*n* = 71)	Aducanumab (*n* = 69)	Donanemab (*n* = 27)	Aducanumab (*n* = 28)
Female, *n* (%)	38 (53.5)	42 (60.9)	14 (51.9)	15 (53.6)
Age, years, mean (SD)	74.1 (6.9)	72.7 (6.8)	76.1 (5.6)	72.9 (6.4)
Race, *n* (%)				
Asian	0 (0.0)	2 (2.9)	0 (0.0)	2 (7.1)
Black or African American	0 (0.0)	5 (7.2)	0 (0.0)	2 (7.1)
White	71 (100)	61 (88.4)	27 (100)	24 (85.7)
Multiple	0 (0.0)	1 (1.4)	0 (0.0)	0 (0.0)
Hispanic/Latino ethnicity, *n* (%)	3 (4.2)	4 (5.8)	0 (0.0)	1 (3.6)
*APOE* genotype, *n* (%)				
ε2/ε3	1 (1.4)	0 (0.0)	0 (0.0)	0 (0.0)
ε2/ε4	2 (2.8)	3 (4.3)	0 (0.0)	1 (3.6)
ε3/ε3	21 (29.6)	20 (29.0)	5 (18.5)	9 (32.1)
ε3/ε4	38 (53.5)	36 (52.2)	18 (66.7)	14 (50.0)
ε4/ε4	9 (12.7)	10 (14.5)	4 (14.8)	4 (14.3)
AChEI use, *n* (%)	28 (39.4)	38 (55.1)	14 (51.9)	15 (53.6)
Memantine use, *n* (%)	13 (18.3)	18 (26.1)	8 (29.6)	7 (25.0)
CDR‐GS, *n* (%)				
0.5	55 (77.5)	47 (68.1)	20 (74.1)	19 (67.9)
1.0	16 (22.5)	22 (31.9)	7 (25.9)	9 (32.1)
MMSE score, mean (SD)	25.0 (2.7)	24.4 (2.9)	24.0 (2.2)	23.9 (2.6)
Amyloid level on PET, CL, mean (SD)	97.2 (29.2)	102.3 (34.6)	104.2 (25.5)	101.8 (28.1)
Tau PET MUBADA SUVR, mean (SD)	1.3 (0.3)	1.3 (0.3)	1.2 (0.1)	1.2 (0.1)

Abbreviations: AChEI, acetylcholinesterase inhibitor; *APOE*, apolipoprotein E; CDR‐GS, Clinical Dementia Rating Global Score; CL, Centiloids; MMSE, Mini‐Mental State Examination; MUBADA, multiblock barycentric discriminant analysis; PET, positron emission tomography; SD, standard deviation; SUVR, standardized uptake value ratio.

### 3.2 Coprimary outcomes

**FIGURE 2 alz70293-fig-0002:**
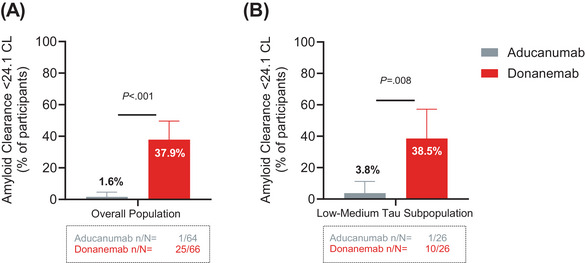
Superiority of donanemab versus aducanumab in AP clearance at 6 months in the (A) overall population and (B) low–medium tau subpopulation. AP, amyloid plaque; CL, Centiloids; *n*, number of participants; *N*, number of participants in the analysis population

AP clearance was defined as < 24.1 CL as assessed by florbetapir PET. In the overall population, 37.9% (25 of 66) of donanemab‐treated participants versus 1.6% (1 of 64) of aducanumab‐treated participants reached AP clearance at 6 months (*P *< 0.001; Figure 2A). In the low–medium tau subpopulation, 38.5% (10 of 26) of donanemab‐treated participants versus 3.8% (1 of 26) of aducanumab‐treated participants reached AP clearance at 6 months (*P *= 0.008; Figure 2B).

### 3.3 Secondary outcomes

#### 3.3.1 AP clearance

**FIGURE 3 alz70293-fig-0003:**
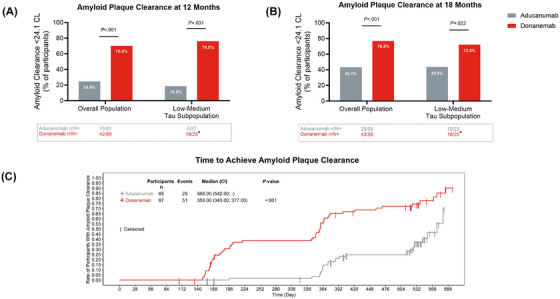
Superiority of donanemab versus aducanumab in AP clearance at (A) 12 months and (B) 18 months, and (C) time to achieve AP clearance. *P* value for (A) and (B) derived from a longitudinal logistic regression model. *P* value for (C): 2‐sided log‐rank unstratified *P* value comparing donanemab versus aducanumab.^*^One participant who achieved amyloid clearance (< 24.1 CL) at week 52 did not meet the threshold for amyloid clearance at week 76. AP, amyloid plaque; CI, confidence interval; CL, Centiloids; LS, least squares; *n*, number of participants; *N*, number of participants in the analysis population

At 12 months, 70.0% (42 of 60) of donanemab‐treated participants and 24.6% (15 of 61) of aducanumab‐treated participants reached AP clearance (*P *< 0.001) in the overall population (Figure 3A). Similarly, at 18 months, 76.8% (43 of 56) of donanemab‐treated participants versus 43.1% (25 of 58) of aducanumab‐treated participants reached AP clearance (*P *< 0.001; Figure 3B). In the low–medium tau subpopulation, 76.0% (19 of 25) and 72.0% (18 of 25) of donanemab‐treated participants versus 18.5% (5 of 27) and 43.5% (10 of 23) of aducanumab‐treated participants reached AP clearance at 12 months (*P *< 0.001) and 18 months (*P *= 0.022).

#### 3.3.2 Amyloid lowering

Absolute and relative[Fig alz70293-fig-0002], [Fig alz70293-fig-0003] amyloid lowering at 6, 12, and 18 months is illustrated in Table 2. The median time to achieve AP clearance was 359 days for donanemab‐treated participants versus 568 days for aducanumab‐treated participants (*P *< 0.001; Figure 3C).

**TABLE 2:** Mean change in amyloid PET from baseline.

	Overall population			Low–medium tau subpopulation		
Months	Donanemab	Aducanumab	*P* value	Donanemab	Aducanumab	*P* value
**6**						
LS mean percent change	−65.17	−16.96	<0.001	−63.91	−25.37	.001
LS mean change (CL)	−62.10	−16.41	<0.001	−64.08	−23.82	<0.001
**12**						
LS mean percent change	−82.79	−56.97	<0.001	−82.74	−56.98	<0.001
LS mean change (CL)	−80.03	−56.06	<0.001	−84.53	−57.77	<0.001
**18**						
LS mean percent change	−86.29	−72.84	.002	−84.64	−70.98	.042
LS mean change (CL)	−84.22	−72.18	.004	−86.57	−72.24	.028

*NOTE*: Percent change in brain AP was defined as the postbaseline PET CL value minus the baseline PET CL value, divided by the baseline PET CL value.

Abbreviations: AP, amyloid plaque; CL, Centiloids; LS, least squares; PET, positron emission tomography.

#### 3.3.3 Biomarker assessments

**FIGURE 4:**
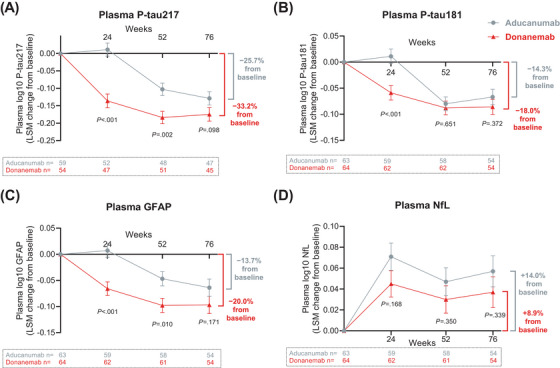
Donanemab and aducanumab treatment demonstrated changes in plasma biomarkers of (A) p‐tau217, (B) p‐tau181, (C) GFAP, and (D) NfL at 6, 12, and 18 months in the overall population. Data are shown as LSM (standard error). GFAP, glial fibrillary acidic protein; LS, least squares mean; *n*, number of participants; NfL, neurofilament light chain; PET, positron emission tomography; p‐tau181, phosphorylated tau 181; p‐tau217, phosphorylated tau 217

In the overall population, compared to baseline levels, plasma phosphorylated tau (p‐tau)217 levels at 18 months were 33.2% lower with donanemab treatment compared to 25.7% lower with aducanumab treatment. The adjusted mean change difference versus aducanumab was −0.148 (95% CI, −0.202 to −0.093; *P *< 0.001) at 6 months, −0.081 (95% CI, −0.132 to −0.030; *P *= 0.002) at 12 months, and −0.046 (95% CI, −0.100 to 0.009; *P *= 0.098) at 18 months (Figure 4A).

Plasma p‐tau181 levels in the overall population were 18.0% lower with donanemab treatment compared to 14.3% lower with aducanumab treatment at 18 months versus baseline. The adjusted mean change difference versus aducanumab was −0.070 (95% CI, −0.109 to −0.030; *P *< 0.001) at 6 months, −0.009 (95% CI, −0.046 to 0.029; *P *= 0.651) at 12 months, and −0.019 (95% CI, −0.060 to 0.023; *P *= 0.372) at 18 months (Figure 4B).

Plasma levels of glial fibrillary acidic protein (GFAP) in the overall population were 20.0% lower with donanemab treatment compared to 13.7% lower with aducanumab treatment at 18 months versus baseline. The adjusted mean change difference versus aducanumab was −0.073 (95% CI, −0.110 to −0.036; *P *< 0.001) at 6 months, −0.051 (95% CI, −0.090 to −0.013; *P *= 0.010) at 12 months, and −0.032 (95% CI, −0.079 to 0.014; *P *= 0.171) at 18 months (Figure 4C).

In the overall population, plasma levels of NfL at 18 months were 8.9% higher with donanemab treatment compared to 14.0% higher with aducanumab treatment versus baseline. The adjusted mean change difference versus aducanumab was −0.025 (95% CI, −0.062 to 0.011; *P *= 0.168) at 6 months, −0.018 (95% CI, −0.055 to 0.020; *P *= 0.350) at 12 months, and −0.020 (95% CI, −0.062 to 0.022; *P *= 0.339) at 18 months (Figure 4D). Biomarker assessment details for the low–medium tau subpopulation are shown in Figure S1 in supporting information.

### 3.4 AEs and tolerability

There were no deaths in this study. The overall incidence of one or more serious AE (SAE) in the donanemab and aducanumab treatment groups was 18.3% (13 of 71) and 11.6% (8 of 69), respectively. Overall, TEAEs were reported for 59 of 71 donanemab‐treated participants (83.1%) and 60 of 69 aducanumab‐treated participants (87.0%), of which 26 (36.6%) in the donanemab group and 32 (46.4%) in the aducanumab group were considered related to study treatment (Table 3).

**TABLE 3:** Summary of AEs.

Event	Donanemab (*n* = 71)	Aducanumab (*n* = 69)
**Overview of AEs, *n* (%)**		
Deaths	0 (0.0)	0 (0.0)
Participants with ≥ 1 SAE	13 (18.3)	8 (11.6)
Treatment discontinuations due to AE	5 (7.0)	4 (5.8)
Study discontinuations due to AE	3 (4.2)	2 (2.9)
TEAEs	59 (83.1)	60 (87.0)
TEAEs related to study treatment	26 (36.6)	32 (46.4)
ARIA‐E or ARIA‐H^a^	21 (29.6)	28 (40.6)
**SAE incidence of ≥2%, *n* (%)**		
ARIA‐E	1 (1.4)^b^	2 (2.9)
Chest pain	0 (0.0)	2 (2.9)
COVID‐19	2 (2.8)	1 (1.4)
Pneumonia	3 (4.2)	0 (0.0)
**TEAE incidence of ≥ 5%, *n* (%)**		
ARIA‐E^a^	17 (23.9)	24 (34.8)
Symptomatic^a^	2 (2.8)	5 (7.2)
ARIA‐H^a^, ^c^	16 (22.5)	23 (33.3)
Symptomatic^a^, ^c^	1 (1.4)	1 (1.4)
Arthralgia	5 (7.0)	2 (2.9)
Chest pain	1 (1.4)	4 (5.8)
COVID‐19	22 (31.0)	14 (20.3)
Dizziness	6 (8.5)	3 (4.3)
Fall	8 (11.3)	5 (7.2)
Headache	8 (11.3)	10 (14.5)
Hypotension	4 (5.6)	0 (0.0)
Infusion‐related reaction (all mild/moderate)	6 (8.5)	2 (2.9)
Nausea	2 (2.8)	4 (5.8)
Superficial siderosis of CNS	0 (0.0)	7 (10.1)
Upper respiratory tract infection	4 (5.6)	3 (4.3)
Urinary tract infection	5 (7.0)	2 (2.9)

^a^ Based on MRI or TEAE cluster.

^b^ Reported as brain edema.

^c^ ARIA‐H includes microhemorrhage and superficial siderosis.

Abbreviations: AE, adverse event; ARIA‐E, amyloid‐related imaging abnormalities of edema/effusion; ARIA‐H, amyloid‐related imaging abnormalities of microhemorrhage and hemosiderin deposits; CNS, central nervous system; MRI, magnetic resonance imaging; SAE, serious adverse event; TEAE, treatment‐emergent adverse event.

The most commonly reported SAEs (≥ 2% incidence in either treatment group) were ARIA‐E (one participant [1.4%] in the donanemab group [reported as brain edema] and two participants [2.9%] in the aducanumab group), chest pain (two participants [2.9%] in the aducanumab group), pneumonia (three participants [4.2%] in the donanemab group), and COVID‐19 (two participants [2.8%] in the donanemab group and one participant [1.4%] in the aducanumab group; Table 3).

The most commonly reported TEAEs among participants in the donanemab and aducanumab groups, respectively, were ARIA‐E (23.9% and 34.8%), COVID‐19 (31.0% and 20.3%), ARIA‐H (22.5% and 33.3%), headache (11.3% and 14.5%), and fall (11.3% and 7.2%; Table 3). The occurrence of infusion‐related reactions was higher in the donanemab group (8.5%) versus the aducanumab group (2.9%), whereas there were seven cases (10.1%) of superficial siderosis in the aducanumab group and none in the donanemab group (Table 3).

**FIGURE 5:**
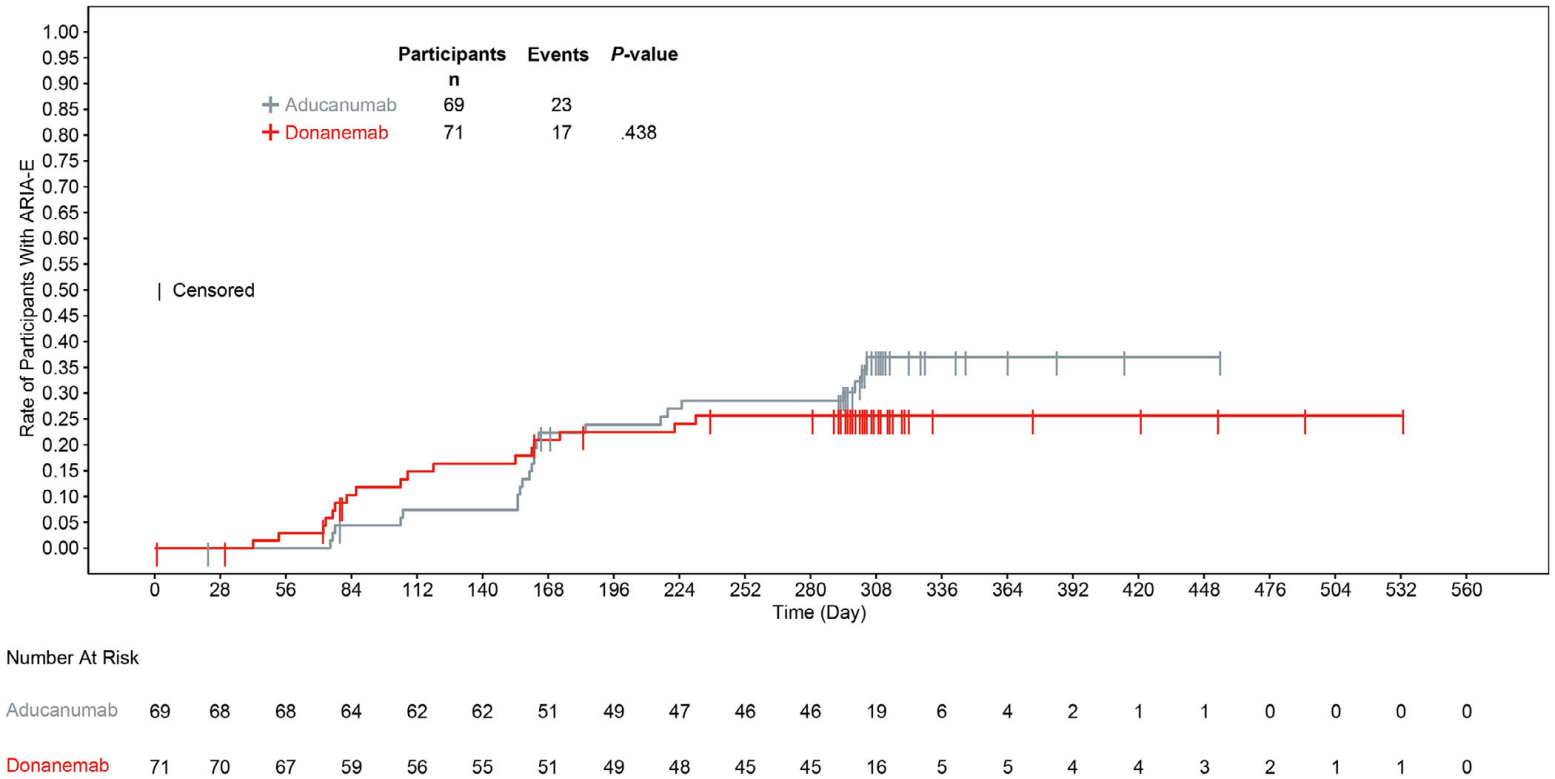
Time to first ARIA‐E based on safety magnetic resonance imaging: Kaplan–Meier analysis. ARIA‐E, amyloid‐related imaging abnormalities of edema/effusion

The incidence of ARIA‐E was higher in the aducanumab group versus the donanemab group. One case (1.4%) of ARIA‐E (based on TEAE cluster) in the donanemab group versus two cases (2.9%) in the aducanumab group were considered serious. Events of ARIA‐E with donanemab and aducanumab were mostly of mild to moderate radiographic severity (88.2% and 82.6% of ARIA‐E events, respectively) based on central MRI readings. Symptomatic ARIA‐E was reported for two participants (2.8%) in the donanemab group and five participants (7.2%) in the aducanumab group (Table 3). Approximately 80% of the first observed ARIA‐E events occurred by weeks 24 and 34 in the donanemab and aducanumab groups, respectively (Figure 5). The incidence of ARIA‐H was higher in the aducanumab group versus the donanemab group, and no serious cases were reported (Table 3). Symptomatic ARIA‐H was reported for one participant (1.4%) each in the donanemab and aducanumab groups (Table 3).

ARIA‐E and ARIA‐H were numerically less common among *APOE* ε4 non‐carriers than among carriers in both treatment groups, with a higher frequency observed among *APOE* ε4 homozygotes than among *APOE* ε4 heterozygotes. One brain macrohemorrhage, which was asymptomatic, occurred in a heterozygous *APOE* ε4 carrier in the aducanumab group (Table S1 in supporting information).

## 4 DISCUSSION

TRAILBLAZER‐ALZ 4 is the first study to directly compare ATTs. Compared to aducanumab‐treated participants, significantly more donanemab‐treated participants met the coprimary endpoints, with a higher proportion of participants reaching AP clearance in the overall population and the low–medium tau subpopulation at 6 months. Similarly, donanemab‐treated participants had a statistically significantly greater reduction in AP levels compared to aducanumab‐treated participants at 12 and 18 months. In addition to these amyloid findings, consistent changes were observed with plasma biomarkers. The incidence of ARIA was consistent with prior reports for each molecule and numerically higher in the aducanumab group, suggesting that the amount and rapidity of amyloid clearance do not necessarily correspond to an increased occurrence of ARIA. These data warrant exploration of novel approaches for implementing ATTs to enhance amyloid removal while reducing ARIA risk.

Moreover, the AP clearance observed in the low–medium tau subpopulation recapitulates the data seen in analogous groups in both the TRAILBLAZER‐ALZ and TRAILBLAZER‐ALZ 2 trials.^13^, ^14^

At 18 months, donanemab showed a greater numerical decrease in plasma p‐tau217, p‐tau181, and GFAP than aducanumab, but did not achieve statistical significance. This is perhaps because plasma biomarker levels tend to be more variable, limiting their statistical power. Additionally, the remaining AD pathology may continue to contribute to the elevation of these plasma biomarkers even after achieving AP clearance (< 24.1 CL). No significant treatment effect on NfL was observed in TRAILBLAZER‐ALZ 4. Further research is needed to understand how treatment with ATTs affects NfL.

TRAILBLAZER‐ALZ 4 had several limitations. The trial was an open‐label study, enabling awareness of treatment allocation for both trial participants and investigators, which may have impacted safety reporting. However, MRI schedules were identical for both treatment groups of the study, and a centrally blinded review of MRI scans and amyloid PET scans were performed. Due to the nature of an open‐label investigation, cognitive and functional measures were not performed, rendering any conclusions of greater or rapid amyloid clearance of unclear clinical significance. However, it is commonly accepted that if a particular drug requires a prolonged period to affect a positive change, then that therapy is less preferable than a drug with a similar safety profile that requires less time to achieve the same effect. Another limitation was that mechanistic differences in therapies may not have been detected by amyloid PET alone. However, no methodology currently exists to measure soluble oligomers and insoluble fibrils. In addition to trial design limitations, the diversity of trial participants remains a challenge in this therapeutic space. There is a need for an improved scale of participant reach, diversity of trial sites and locations, improved community outreach and retention, and a better understanding of the variables for screening failures for interested participants.^23^ Finally, the clinical relevance of this comparison is uncertain given that aducanumab is no longer available. However, at the time of conception and design of TRAILBLAZER ALZ 4 it was anticipated aducanumab would become the standard of care for early symptomatic AD.

TRAILBLAZER‐ALZ 4 is the first study to directly compare ATTs. In this phase 3 trial in participants with early symptomatic AD, donanemab demonstrated superiority over aducanumab in AP clearance in both the overall population and the low–medium tau subpopulation. Moreover, the amount and speed at which AP was cleared did not result in an increased frequency and/or risk of ARIAs. Ongoing studies are assessing the impact of donanemab in AP removal in preclinical AD and exploring different dosing paradigms. The effect of donanemab in clinical practice will continue to be evaluated and monitored.

## AUTHOR CONTRIBUTIONS

Conception: Andrew Pain, Margaret B. Ferguson, Ena Oru. Design of the work: Andrew Pain, Margaret B. Ferguson, Hong Wang, Ena Oru, Emily C. Collins, Dawn A. Brooks, John R. Sims. Acquisition of data: Stephen Salloway, Andrew Pain, Elly Lee, Michelle Papka, Margaret B. Ferguson, Hong Wang, Paul A. Ardayfio, Emily C. Collins. Analysis of data: Stephen Salloway, Andrew Pain, Elly Lee, Hong Wang, Haoyan Hu, Paul A. Ardayfio, Deirdre B. Hoban, Emily C. Collins, Dawn A. Brooks, John R. Sims. Interpretation of data: Stephen Salloway, Andrew Pain, Margaret B. Ferguson, Hong Wang, Haoyan Hu, Ming Lu, Paul A. Ardayfio, Deirdre B. Hoban, Emily C. Collins, Dawn A. Brooks, John R. Sims. Drafting of the work: Andrew Pain, Hong Wang, Haoyan Hu, Ena Oru, Paul A. Ardayfio, Deirdre B. Hoban. Critical revision of the work for important intellectual content: Stephen Salloway, Andrew Pain, Elly Lee, Michelle Papka, Margaret B. Ferguson, Hong Wang, Haoyan Hu, Ming Lu, Paul A. Ardayfio, Deirdre B. Hoban, Emily C. Collins, Dawn A. Brooks, John R. Sims. All authors provided input and gave final approval for the work to be published.

## CONFLICT OF INTEREST STATEMENT

Stephen Salloway: Butler Hospital receives research support for conducting clinical trials through grants or contracts from Biogen, Eisai, Genentech, Roche, CognitionRx, Eli Lilly and Company, and Janssen. He has received consulting fees from Abbvie, Acumen, Alector, Biogen, Biohaven, Cognition, Eisai, Fujirebio, Genentech, Kisbee, Laqbcorp, Lilly, Merck, Neurophet, NovoNordisk, Prothena, Quest, and Roche. Dr. Salloway is an author for the Appropriate Use Recommendations for aducanumab, lecanemab, and donanemab. Elly Lee: Irvine Clinical research receives study‐related funds from Eli and Company, Eisai, Biogen, Prothena, Abbvie, BMS, Janssen, Acumen, Roche, and Neumora. Michelle Papka received consulting fees from Eli Lilly and Company. Andrew Pain, Margaret B. Ferguson, Hong Wang, Haoyan Hu, Ming Lu, Ena Oru, Paul A. Ardayfio, Deirdre B. Hoban, Emily C. Collins, Dawn A. Brooks, and John R. Sims are employees of and minor shareholders of Eli Lilly and Company. Author disclosures are available in the supporting information.

## CONSENT STATEMENT

All participants provided written informed consent before the start of the study.

## Supporting information



Supporting Information

Supporting Information

## Data Availability

Lilly provides access to all individual participant data collected during the trial, after anonymization, with the exception of pharmacokinetic or genetic data. Data are available to request 6 months after the indication studied has been approved in the United States and EU and after primary publication acceptance, whichever is later. No expiration date of data requests is currently set once data are made available. Access is provided after a proposal has been approved by an independent review committee identified for this purpose and after receipt of a signed data sharing agreement. Data and documents, including the study protocol, statistical analysis plan, clinical study report, and blank or annotated case report forms, will be provided in a secure data sharing environment. For details on submitting a request, see the instructions provided at www.vivli.org.
