# Supplementary material for: TRAILBLAZER‐ALZ 4: A phase 3 trial comparing donanemab with aducanumab on amyloid plaque clearance in early, symptomatic Alzheimer's disease
Source: Alzheimers Dement. 2025 May 19;21(5):e70293. doi: 10.1002/alz.70293 (PMC12089073; doi:10.1002/alz.70293)
Supplement: Supplementary file 1 — Supporting Information [file ALZ-21-e70293-s001.pdf]

SUPPLEMENTARY MATERIAL

Supplement 1: Additional Tables and Figures

SUPPLEMENTARY TABLE 1 ARIA and macrohemorrhage by *APOE* ε4 genotype at 18 months.

	Aducanumab (N=69)			Donanemab (N=71)		
	APOE ε4 genotype					
Event n (%)	Noncarrier (N=20)	Heterozygote (N=39)	Homozygote (N=10)	Noncarrier (N=22)	Heterozygote (N=40)	Homozygote (N=9)
ARIA-E or ARIA-H*	7 (35.0)	14 (35.9)	7 (70.0)	3 (13.6)	14 (35.0)	4 (44.4)
ARIA-E*	5 (25.0)	12 (30.8)	7 (70.0)	2 (9.1)	11 (27.5)	4 (44.4)
Symptomatic	1 (5.0)	2 (5.1)	2 (20.0)	0 (0.0)	2 (5.0)	0 (0.0)
SAE†	1 (5.0)	1 (2.6)	0 (0.0)	1 (4.5)	0 (0.0)	0 (0.0)
ARIA-H*,‡,§	5 (25.0)	13 (33.3)	5 (50.0)	2 (9.1)	10 (25.0)	4 (44.4)
Symptomatic*,‡	1 (5.0)	0 (0.0)	0 (0.0)	0 (0.0)	1 (2.5)	0 (0.0)
Macrohemorrhage*,§	0 (0.0)	1 (2.6)	0 (0.0)	0 (0.0)	0 (0.0)	0 (0.0)

* Based on MRI or TEAE cluster; † Based on TEAE cluster; ‡ ARIA-H includes microhemorrhage and superficial siderosis; § There were no SAE cases for ARIA-H or macrohemorrhage.

Abbreviations: *APOE*, apolipoprotein E; ARIA-E, amyloid-related imaging abnormalities of edema/effusion; ARIA-H, amyloid-related imaging abnormalities of microhemorrhage and hemosiderin deposits; MRI, magnetic resonance imaging; SAE, serious adverse event; TEAE, treatment-emergent adverse event.

SUPPLEMENTARY FIGURE 1 Donanemab and aducanumab treatment demonstrated changes in plasma biomarkers of (A) GFAP, (B) P-tau217, (C) P-tau181, and (D) NfL at 6, 12, and 18 months in the low-medium tau subpopulation.

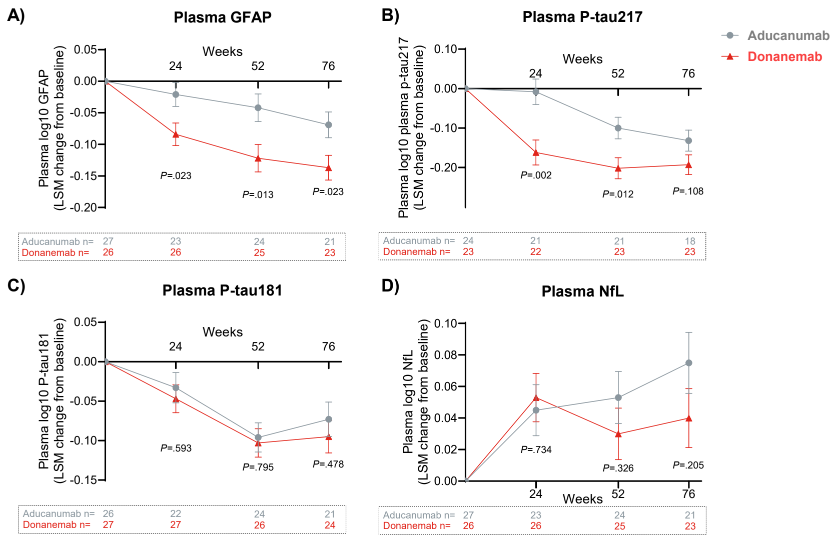

Data are shown as LSM (standard error). *P*-value represents treatment difference of donanemab vs aducanumab.

Abbreviations: GFAP, glial fibrillary acidic protein; LSM, least-squares mean; n, number of participants; NfL, neurofilament light chain; P-tau217, phosphorylated tau 217; P-tau181, phosphorylated tau 181.
